# Association between Large Arteries Diameter and Heart Function in Subjects Free of Cardiovascular Diseases

**DOI:** 10.3390/jpm12060889

**Published:** 2022-05-28

**Authors:** Ricarda von Krüchten, Roberto Lorbeer, Annette Peters, Fabian Bamberg, Christopher L. Schlett, Blerim Mujaj

**Affiliations:** 1 Department of Diagnostic and Interventional Radiology, Medical Center, Faculty of Medicine Freiburg, University of Freiburg, Hugstetter Str. 55, 79106 Freiburg, Germany; ricarda.kruechten@uniklinik-freiburg.de (R.v.K.); fabian.bamberg@uniklinik-freiburg.de (F.B.); Christopher.schlett@uniklinik-freiburg.de (C.L.S.); 2 Department of Radiology, Ludwig-Maximilians-University Hospital, Marchioninistraße 15, 81377 Munich, Germany; roberto.lorbeer@med.uni-muenchen.de; 3 Chair of Epidemiology, Institute for Medical Information Processing, Biometry and Epidemiology, Medical Faculty, Ludwig-Maximilians-University Hospital, Marchioninistraße 15, 81377 Munich, Germany; peters@helmholtz-muenchen.de; 4 Institute of Epidemiology, German Research Center for Environmental Health, Helmholtz Zentrum München, Ingolstädter Landstr. 1, 85764 Neuherberg, Germany; 5 German Center for Diabetes Research (DZD), Partner Site Neuherberg, Ingolstädter Landstr. 1, 85764 Neuherberg, Germany

**Keywords:** aortic diameter, pulmonary artery diameter, ratio pulmonary artery/aorta diameter, cardiac function parameters, magnetic resonance imaging, population-based cohort

## Abstract

To investigate the association between Aorta (Ao), pulmonary artery (PA) diameters and the PA/Ao ratio with right (RV) and left ventricle (LV) volumetric properties in subjects free of cardiovascular diseases. In the KORA-MRI study, 339 subjects (mean age 56.3 ± 9.1 years; 43.7% female) underwent whole-body 3T-MRI. Ao and PA were measured on DIXON sequences. Cvi42 quantified cardiac functional parameters from a SSFP sequence. The relationship between ascending (AAo), and descending aorta (DAo), as well as PA diameters, and RV and LV function were assessed using linear regression models adjusted for age, sex, and cardiovascular risk factors. AAo and DAo diameter were associated with LV end-diastolic volume (β = 4.52, *p* = 0.015; ß = 7.1, *p* ≤ 0.001), LV end-systolic volume (β = 2.37, *p* = 0.031; ß = 3.66, *p* = 0.002), while DAo associated with RV end-diastolic volume (β = 6.45, *p* = 0.006) and RV end-systolic volume (β = 3.9, *p* = 0.011). PA diameter was associated with LV end-diastolic volume (β = 4.81, *p* = 0.003). Interestingly, the PA/Ao ratio was only associated with RV end-diastolic and end-systolic volume (β = 4.48, *p* = 0.029; ß = 2.82, *p* = 0.037). Furthermore, we found different relationships between men and women. Ao and PA diameter were associated with LV and RV volumetric parameters in subjects free of cardiovascular diseases suggesting that ventricular volumetric performance directly relates to vascular diameter properties.

## 1. Introduction

Left ventricular (LV) performance is the primary determinant of cardiac function, which may be influenced by arterial properties [1,2]. Understanding the performance of LV requires not only the examination of the LV itself but also assessment of the right ventricle (RV) and effects of the arterial system, mainly the aorta (Ao) and pulmonary artery. Structural changes in the arterial system may affect cardiac physiology and pathology. Enlargement of ascending aorta (AAo) diameter is a frequent finding in clinical practice and enlarged main pulmonary artery (MPA) and the ratio of MPA/Ao are indirect signs of pulmonary hypertension. In contrast, the AAo diameter is known to increase with age [3]. The increase in aortic diameter is associated with remodeling the aortic wall tissue, which includes rupture of elastin fibers, increased collagen production, and calcification, all of which are related to aortic wall stiffness [4,5]. Besides age, smoking [6], elevated pressure [7], i.e., hypertension or bicuspid aortic valve leaflets [6], and disorders that weaken the aorta wall, such as atherosclerosis [7], Marfan’s disease, and other connective tissue diseases [8] are all known risk factors for aortic dilatation and lead to aortic aneurysm. Moreover, studies suggest that MPA/Ao diameter ratio is an essential predictor of pulmonary hypertension and chronic obstructive pulmonary disease (COPD) exacerbations [9]. Further, a ratio of MPA/Ao of 1.0 or higher was related to cardiovascular events in patients referred for cardiac magnetic resonance imaging (MRI). Changes in large vessels diameter generally occur before cardiac dysfunction and remain asymptomatic unless a significant cardiac function impairment occurs and further investigation is needed. However, it remains unclear whether cardiac function plays a role in aortic and pulmonary artery diameters and how their diameter is related directly to LV and RV functional performance. To date, MRI enables heart and large arteries imaging with excellent structural and functional visualization to understand the relationship between large artery diameter and ventricular function. Against this background, we investigated the association between the Ao diameter and diameters of the pulmonary artery and LV and RV function in participants free of cardiovascular diseases (CVD) in the KORA-MRI study.

## 2. Materials and Methods

### 2.1. Study Population

The KORA-MRI study enrolled participants aged 25 to 74 years old from the Augsburg region of Germany [10]. Between June 2013 and September 2014, participants were assessed at the KORA study center, and a 3 Tesla whole-body MRI scan was done [10]. Being in the prediabetes, diabetes, or control group and written informed consent from all individuals were required to participate in the whole-body MRI scan. Exclusion criteria were: age > 74 years, validated stroke or myocardial infarction, arterial vessel occlusion, MRI contraindications (pregnancy, breastfeeding, large-area tattoo (palm of hand) or old (>20 years) tattoo, cardiac pacemaker, stents, heart defibrillator, other metal parts inside the body, impaired renal function, serum creatinine ≥ 1.3 mg/dL, claustrophobia, gadolinium allergy, one-hour supine position or 15 sec. breathing pause not possible), poor overall health condition. The Institutional Research Ethics Board of the Medical Faculty of Ludwig-Maximilian University Munich approved the KORA-MRI study, which followed the Helsinki Declaration on Human Research [11].

### 2.2. Whole-Body MR Imaging Protocol

The whole-body MRI scans were performed using a 3-Tesla MRI system (MagnetomSkyra, Siemens AG, Healthcare Sector, Erlangen, Germany) [10] with an 18-channel body surface coil and a table-mounted spine matrix coil. The technique includes sequences covering the full body from neck to head of femur for specific organs, such as the brain, carotid arteries, tissue/organ quantification, and fat compartments. In axial slice orientation, the thoracic aortic diameter and pulmonary artery diameters were measured using two-point gradient-echo Dixon vibe sequence images. Imaging parameters were: voxel size 1.5 × 1.5 mm^2^, slice thickness (ST) 3 mm, field of view (FOV) 297 × 360 mm, matrix 320 × 195, repetition time/echo time (TR/TE) 4.1/1.23; 2.46; 3.69; 4.92; 6.15; 7.38, flip angle (FA) 9°. For analysis of the cardiac parameters, the cine-steady-state free precession sequence was obtained in a short-axis perspective with 10 layers and 25 phases. A FOV of 297 mm × 360 mm, a matrix of 240 × 160, a TR of 29.97 ms, a TE of 1.46 ms, and a FA of 62° were all included in the 8 mm slice thickness.

### 2.3. MR-Image Analysis for Measurement of Vessel Diameters

**Figure 1:**
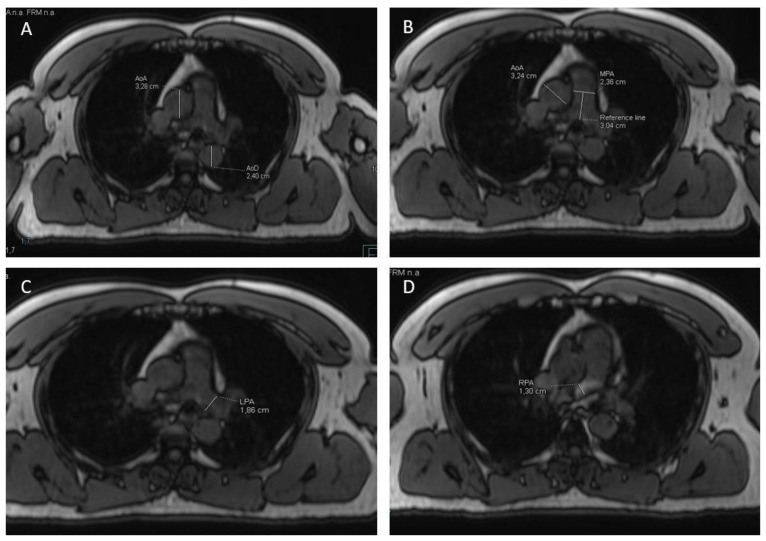
Measurement of diameters of aorta and pulmonary artery. (**A**): Measurement of ascending (AoA) and descending aorta (AoD) at the level of the right pulmonary artery (RPA) in anterior-posterior orientation. (**B**): Measurement of main pulmonary artery (MPA) orthogonally to the reference line at the level of the pulmonary bifurcation. Reference line drawn along the course of the vessel of ca. 3 cm (±0.1 cm). Diameter of AoA determined on same image for determination of MPA/Ao-ratio. (**C**,**D**): Measurement of left pulmonary artery (LPA) and RPA measured at its maximum diameter on axial images.

The outer diameters were measured at two predefined aortic segments: AAo and descending aorta (DAo) at the level of the right pulmonary artery (RPA) (Figure 1). Diameters were measured on axial slices in anterior–posterior orientation from outer walls using Syngo.via (Siemens, Healthcare, Erlangen, Germany). To determine the diameter of the MPA, the height of the pulmonary artery branch was identified individually on the axial images, and a reference line of approximately 3 cm (±0.1 cm) was drawn along the course of the vessel (Figure 1) according to prior validated CT protocols [12,13]. The vessel diameter is measured orthogonal to the reference line. To define the MPA/Ao ratio, the diameter of the ascending aorta was determined on the same image as the MPA. The cut-off value for MPA/Ao was 1.0. This number has been demonstrated to be specific to most CT investigations as an upper cutoff value [14]. To assess the diameter of the RPA and left pulmonary artery (LPA), vessels were identified and measured at their maximum diameter on axial images. Diameters were also measured using Syngo.via (Siemens, Healthcare, Erlangen, Germany).

### 2.4. MR-Image Analysis for Cardiac Measurements

Cine steady-state free precession (cine-SSFP) sequences were performed for imaging of heart function, morphology, and cardiac morphology disorders. The LV and RV function was assessed using Circle Cardiovascular Imaging Inc.’s cvi42 software (version 4.1.5 (190), Calgary, Alberta, Canada). The detection of LV contours and calculation of LV volumes were done automatically, then manually corrected, if necessary [15], and the LV myocardial mass was measured during end-diastole. The software computed the corresponding volumes after manually segmenting the RV lumen in end-systole and end-diastole in each layer from the cardiac apex to the pulmonary valve [16]. The parameters for stroke volume and ejection fraction are composed of the difference between the end-systolic and end-diastolic volumes. pyHeart, dedicated in-house software that displays LV time-volume curves, was also used to quantify LV filling and ejection rates [17,18]. Peak gradients were predicted during systolic ejection, early LV filling (which is largely passive), and late LV filling (which is powered by atrial contraction) [19].

### 2.5. Assessment of Risk Factors

Physical examination, interview, and blood samples were used to collect information on risk factors. Body mass index (BMI) and body surface area (BSA) were determined using height and weight, and smoking status, as well as antihypertensive and lipid-lowering medicine (statin usage), were evaluated using a questionnaire. Systolic blood pressure > 140 mmHg, diastolic blood pressure > 90 mmHg, or current antihypertensive therapy were all considered hypertension. Prediabetes (Impaired fasting glucose: average fasting glucose concentration and a 2-h serum oral glucose tolerance test (OGTT) glucose concentration between 140 and 200 mg/dL; and/or impaired fasting glucose concentration, as defined by fasting glucose levels between 110 and 125 mg/dL and an average 2-h serum glucose concentration), and diabetes (2-h serum glucose concentration as determined by OGTT that was >200 mg/dL and/or a fasting glucose concentration between 110 and 125 mg/dL) were defined according to the WHO criteria [20]. At the time of the assessment, all study participants’ venous blood was drawn and sent to the Augsburg Central Hospital laboratory within 2 to 4 h. The Boehringer CHOD-PAP (Roche Diagnostics) test determined total cholesterol. Triglycerides were measured with the Boehringer GPO-PAP assay (non-fasting in diabetic subjects) [10].

### 2.6. Statistical Analysis

For continuous and categorical variables, the distribution of demographic characteristics was reported using mean and standard deviation (SD), median (interquartile ranges (IQRs)), or percentages, respectively. We employed a multi-step approach, first focusing on the relationship between the diameter of AAo and ADo, and LV and RV functional characteristics using linear regression models. We adjusted for sex and age in model 1. Smoking, BMI, systolic blood pressure, diastolic blood pressure, and diabetes mellitus were additionally adjusted in Model 2. Model 3 was also adjusted for cholesterol, triglycerides, hypertension medication, and lipid-lowering medication. Second, using the above-mentioned linear regression models, we investigated the relationship between MPA, RPA as well as LPA, the ratio of MPA/Ao, and RV and LV function characteristics. Third, using the same models as in linear regression analysis, stratified analyses for sex were carried out. Stata was used to conduct all analyses (Stata 16.1 Corporation, College Station, TX, USA).

## 3. Results

Table 1 summarizes the study population’s characteristics, including a mean age of 56.3 (±9.1) years and 43.7% female subjects. The mean BMI was 28.0 (±4.8), and the mean BSA was 1.95 (±0.22). 43% of subjects were former smokers, 21% were current smokers, and 36% were never smokers. Overall, the mean diameter of the AAo was 2.97 (±0.40), and of the ADo was 2.09 (±0.31). The mean measurement of the MPA was 2.67 (±0.34), and the ratio of MPA/Ao was 0.91 (±0.15). Cardiac measurements were within expected ranges (Table 1). Clinical history of cardiovascular disease was not present according to inclusion and exclusion criteria.

### 3.1. Association between Diameter of the Aorta and RV and LV Function Parameters

There were significant associations between ascending AAo and ADo diameters and LV and RV functional parameters. AAo and ADo diameter were positively associated with LV end-diastolic volume, end-systolic volume, stroke volume, peak ejection rate, and mass (Table 2, Figure S1). In contrast, only the ADo diameter was positively associated with RV end-diastolic, end-systolic, and stroke volume (Table 2, Figure S1). The association between the AAo and ADo, and LV and RV did not attenuate after adjustment for risk factors in model 2 and model 3 (Table 2, Figure S1).

### 3.2. Association between Diameters of the Pulmonary Artery System and RV and LV Function Parameters

The diameters of the pulmonary artery system were significantly related to LV and RV functional characteristics. The diameter of the MPA was positively associated with LV end-diastolic volume, stroke volume, peak ejection rate, early diastolic rate, late diastolic rate, mass, as well as with RV end-diastolic volume, end-systolic volume, and stroke volume, but not with LV or RV ejection fraction (Table 3, Figure S1). The diameter of the RPA was positively associated with LV end-diastolic volume, stroke volume, peak ejection rate, early and late diastolic rate, mass, as well as with RV end-diastolic volume, end-systolic volume, and stroke volume, but not with LV or RV ejection fraction (Table 3, Figure S1). The diameter of the LPA was positively associated with LV end-diastolic volume, and additionally with end-systolic volume, stroke volume, peak ejection rate, early and late diastolic rate, mass, as well as with RV end-diastolic volume, end-systolic volume, and stroke volume, and not with LV or RV ejection fraction (Table 3, Figure S1). The ratio of MPA/Ao was only positively associated with RV end-diastolic and end-systolic volume (Table 4, Figure S1).

### 3.3. Stratified Analysis According to Sex

In females (Table S1), the diameter of AAo was positively associated with LV mass, while in males, the diameter of AAo and ADo was associated with LV end-diastolic volume, end-systolic volume, and mass (Table S4). The diameter of ADo in males was inversely associated with LV ejection fraction and positively associated with RV end-diastolic volume and end-systolic volume (Table S4).

In females (Table S2), the diameter of the MPA was positively associated with LV end-diastolic and end-systolic volume, stroke volume, peak ejection rate, early diastolic rate, mass, as well as with RV end-diastolic volume, end-systolic volume, and stroke volume, but not with LV or RV ejection fraction. The diameter of the RPA was positively associated with only LV stroke volume, as well as with RV end-diastolic volume, end-systolic volume, and stroke volume. In contrast, the diameter of the LPA was positively associated with LV end-diastolic and end-systolic volume, stroke volume, early diastolic rate, mass, as well as with RV end-diastolic volume, end-systolic volume, and stroke volume, but not with LV or RV ejection fraction (Table S2). In females, the ratio of MPA/Ao was positively associated with LV end-diastolic volume, stroke volume, early diastolic rate, as well as with RV end-diastolic volume, end-systolic volume, and stroke volume (Table S3).

In males (Table S5), the diameter of the MPA was solely positively associated with LV late diastolic rate. The diameter of the RPA was positively associated with LV end-diastolic volume, mass, as well as with RV end-diastolic volume, end-systolic volume, and stroke volume. In opposite, the diameter of the LPA was positively associated with LV end-diastolic volume, stroke volume, peak ejection rate, early and late diastolic rate, as well as with RV end-diastolic volume, end-systolic volume, and stroke volume. The ratio of MPA/Ao was solely positively associated with LV mass (Table S6).

## 4. Discussion

In this sample with subjects free of cardiovascular diseases, our results indicate that larger AAo and ADo diameters were positively associated with LV volumetric parameters, while the ADo diameter was associated with RV volumes. Furthermore, the MPA was positively associated with LV and RV volumetric parameters, while the ratio of MPA/Ao was related to RV end-diastolic and end-systolic volumes.

To date, our study is the first to evaluate the relationship between the aorta and pulmonary artery segments and cardiac volumes in a healthy cohort without established cardiovascular disease. Previously, the data from the CARDIA study, based on echocardiography assessments with a follow-up of 20 years, showed that increased aortic root seize was associated with increased LV mass and concentric LV remodeling in subjects from early adulthood to middle age, confirming a direct link between large artery diameter and cardiac performance [21]. Our study assessed different segments of the aorta, namely ascending and descending, and both segments were significantly related to LV, although estimates between AAo and ADo differed. This might indicate that more distant segments, i.e., ADo diameter variations or pathologies play a vital role in the volumetric performance of both the LV and RV. While this difference between AAo and ADo was observed on volumetric parameters, the same was not observed on LV mass, suggesting that diameter differences influence functional performance more than structural remodeling. A similar difference was observed when assessing the segments of MPA with LV and RV, i.e., LPA and RPA, with higher estimates for LPA compared to RPA. There were no differences between MPA and RPA, but apparent differences between MPA and LPA. It seems that these differences generate from angulation of MPA bifurcation into LPA and RPA, given that the mean diameter between LPA and RPA differs by less than 5%. Previously, AAo segment was elaborated within Framingham Heart Study, in which aortic root size remodeling was associated with an increased risk of heart failure in middle-aged and older individuals [22]. However, increased LV filling pressures with predominant but not isolated diastolic dysfunction described in the pathogenesis of HFpEF subpopulation, beyond heart pathologies, may be explained by unfavorable vascular-ventricular interactions [23]. Our study extends this aspect further by exploring the role of vascular diameters on the volumetric performance of the heart.

Several risk factors influence the vascular system, affecting various conditions [24]. The previous population-based MESA study assessed many risk factors contributing to aortic diameter associated with hypertension and diabetes [25]. Another MESA study evaluated the effect of aorta dilation in smokers by the presence of COPD, emphysema, and airway thickening. A larger aorta diameter in people with COPD and severe emphysema than COPD linked to chronic bronchitis or bronchial wall thickening was identified [25]. Also, emphysema is related to increasing aortic diameters and aortic aneurysms [26]. In addition, we previously assessed the role of insulin and glucose on lung and RV volumes and found that higher insulin levels were associated with lower lung volumes and reduced volumetric RV performance [17].

The link between aorta diameter and LV volumes has been assessed in an athletes’ population, and the study reported a significant relationship between ascending aorta dilatation and LV performance [27]. Our findings are in line with this study which suggests a strong relationship between ascending aorta segment and heart. However, our study builds novel knowledge on the relationship of the heart with other vascular segments such as ADo, and pulmonary artery.

The diameter of MPA, and its relation to the diameter of AAo has been used to identify patients at risk [28] and was associated with cardiovascular outcomes, including pulmonary hypertension [14,29]. In a previous study, MPA/Ao ratio greater than 1.0 was independently associated with increased risk for cardiovascular events, including heart failure and pulmonary hypertension. Regarding the pulmonary artery, our findings reveal the influence on the volumetric performance of RV, including end-diastole and end-systole compared to LV, where the diameter of MPA is associated with end-diastole only. The same observation has been shown with the MPA/Ao ratio, suggesting that a greater ratio than 1.0 affects more RV than LV.

The major strength of the study incorporates the implementation of advanced 3T-whole-body MRI technology with a detailed protocol. Whole-body MRI allows simultaneous assessment of the heart, great vessels, and lungs at a single time point for reliable real-time assessment of cardiopulmonary function. In addition, automated algorithms for MR image analysis have been used to quantify cardiac functional volume. With the increasing establishment of machine learning, whole-body MRI may become increasingly attractive as a one-stop-shop model, especially with regard to personalized medicine. Furthermore, multi-level adjustment was applied to confirm the results. The study recruited healthy individuals free of cardiovascular diseases from a population-based cohort. Nonetheless, there are limitations necessary to mention. First, although the MRI protocol included atria, the atria size or function was not used in adjustment. Second, cross-sectional design study limits to draw causal inferences, and generated hypotheses require further confirmation in other designs and populations. Third, the study represents a relatively small number of participants from the population-based cohort. Fourth, the generalizability of findings may be limited to other populations or geographical regions.

## 5. Conclusions

In conclusion, larger aorta and pulmonary artery diameters were positively associated with LV and RV volumetric parameters in subjects free of cardiovascular diseases suggesting that ventricular volumetric performance directly relates to vascular diameter properties. Further studies with longitudinal designs are needed to confirm our findings.

## Figures and Tables

**Table 1:** Study population characteristics.

	All	Female	Male	*p* Value
	*n* = 339	*n* = 148	*n* = 191	
Age, years	56.3 (±9.1)	56.1 (±9.1)	56.4 (±9.1)	0.695
Women	148 (43.7%)	148 (100%)	0 (0%)	-
Body mass index, kg/m^2^	28.0 (±4.8)	27.6 (±5.5)	28.3 (±4.2)	0.147
Body surface area, m^2^	1.95 (±0.22)	1.79 (±0.17)	2.07 (±0.17)	<0.001
Smoking status				0.153
Never (%)	123 (36.3%)	61 (41.2%)	62 (32.5%)	
Past (%)	145 (42.8%)	55 (37.2%)	90 (47.1%)	
Current (%)	71 (20.9%)	32 (21.6%)	39 (20.4%)	
Alcohol use, (g/day)	17.9 (±21.9)	8.2 (±11.9)	25.3 (±24.8)	<0.001
Physical activity	209 (61.7%)	98 (66.2%)	111 (58.1%)	0.128
Diabetes status				0.016
Normal (%)	210 (62%)	104 (70.3%)	106 (55.5%)	
Prediabetes (%)	88 (26%)	32 (21.6%)	56 (29.3%)	
Diabetes (%)	41 (12.1%)	12 (8.1%)	29 (15.2%)	
Serum glucose, mmol/L	5.5 (5.11; 6.05)	5.27 (4.88; 5.83)	5.61 (5.27; 6.16)	<0.001
Serum insulin, pmol/L	54.6 (37.2;81.0)	49.2 (34.8;74.8)	60 (38.4;90.0)	0.003
Hypertension (%)	112 (33%)	39 (26.4%)	73 (38.2%)	0.021
Systolic, mm/Hg	120.7 (±16.9)	113.2 (±14.5)	126.4 (±16.4)	<0.001
Diastolic, mm/Hg	75.3 (±10.1)	72.2 (±8.6)	77.8 (±10.4)	<0.001
Antihypertensive medication (%)	82 (24.2%)	37 (25%)	45 (23.6%)	0.759
eGFR, ml/min/1.73 m^2^	87.2 (±13.1)	85.9 (±13.2)	88.2 (±13.0)	0.110
Total cholesterol, mmol/L	5.62 (±0.96)	5.65 (±0.91)	5.6 (±0.99)	0.630
HDL, mmol/L	1.61 (±0.46)	1.82 (±0.46)	1.44 (±0.38)	<0.001
LDL, mmol/L	3.61 (±0.86)	3.52 (±0.83)	3.68 (±0.88)	0.102
Triglycerides, mmol/L	1.20 (0.87; 1.75)	1.08 (0.78; 1.37)	1.39 (0.99; 2.13)	<0.001
Lipid lowering medication (%)	36 (10.6%)	16 (10.8%)	20 (10.5%)	0.920
Diameter of				
Ascending aorta (cm)	2.97 (±0.40)	2.80 (±0.36)	3.11 (±0.37)	<0.001
Descending aorta (cm)	2.09 (±0.31)	1.96 (±0.28)	2.20 (±0.29)	<0.001
Main Pulmonary Artery (cm)	2.67 (±0.34)	2.67 (±0.35)	2.68 (±0.33)	0.705
Right pulmonary artery (cm)	1.84 (±0.27)	1.79 (±0.26)	1.87 (±0.27)	0.008
Left pulmonary artery (cm)	1.94 (±0.24)	1.91 (±0.24)	1.97 (±0.24)	0.033
PO/AO	0.91 (±0.15)	0.97 (±0.15)	0.87 (±0.13)	<0.001
Cardiac parameters				
LV End-diastolic Volume, (mL/m^2^)	130.1 (±32.6)	118.3 (±25.9)	139.3 (±34.4)	<0.001
LV End-systolic Volume, (mL/m^2^)	41.1 (±18.2)	35 (±14.8)	45.9 (±19.2)	<0.001
LV Stroke Volume, (mL/m^2^)	89.0 (±20.2)	83.3 (±16.7)	93.5 (±21.6)	<0.001
LV Ejection fraction, (%)	69.2 (±7.8)	71.0 (±6.7)	67.8 (±8.3)	<0.001
LV Peak ejection rate, (mL/s)	356.2 (±134)	333.9 (±107)	373.4 (±149.6)	0.007
LV Early diastolic filling rate, (mL/s)	228.9 (±115.6)	231.4 (±104.8)	227 (±123.6)	0.732
LV Late diastolic filling rate, (mL/s)	240.7 (±142.7)	236.4 (±135)	244.1 (±148.7)	0.623
LV Mass, diastolic, g	140.4 (±35.1)	114.1 (±24.1)	160.8 (±27.9)	<0.001
RV End-diastolic Volume, (mL/m^2^)	164.9 (±39.8)	144.3 (±31.1)	180.9 (±38.6)	<0.001
RV End-systolic Volume, (mL/m^2^)	79.1 (±25.8)	64.5 (±19.1)	90.4 (±24.7)	<0.001
RV Stroke Volume, (mL/m^2^)	85.9 (±19.8)	79.9 (±17.2)	90.6 (±20.4)	<0.001
RV Ejection fraction, (%)	52.7 (±7.1)	55.7 (±6.3)	50.3 (±6.8)	<0.001

The values represent mean ± standard deviation (SD), median (interquartile ranges) or frequency along with percentage (%). *p* = *p*-value for difference (*t*-test, Mann-Whitney-U test or chi2-test); Abbreviation: AO = aorta diameter; CVD = cardiovascular disease, eGFR = estimated glomerular filtration rate, HDL = high-density lipoprotein, LDL = low-density lipoprotein; LV = left ventricle; PO = pulmonary artery diameter; RV = right ventricle.

**Table 2:** Association between Diameter of the aorta and ventricle function parameters.

Diameter Per SD	Model 1	*p* Value	Model 2	*p* Value	Model 3	*p* Value
	LV End–diastolic Volume					
AAo	5.13 (1.52; 8.74)	0.005	5.06 (1.43; 8.68)	0.006	4.52 (0.89; 8.15)	0.015
DAo	7.52 (3.62; 11.4)	<0.001	7.20 (3.3; 11.1)	<0.001	7.1 (3.22; 10.9)	<0.001
	LV End–systolic Volume					
AAo	2.55 (0.48; 4.62)	0.016	2.39 (0.26; 4.51)	0.028	2.37 (0.22; 4.52)	0.031
DAo	3.82 (1.58; 6.06)	0.001	3.68 (1.39; 5.97)	0.002	3.66 (1.35; 5.97)	0.002
	LV Stroke Volume					
AAo	2.57 (0.26; 4.87)	0.030	2.65 (0.36; 4.94)	0.023	2.13 (−0.13; 4.4)	0.065
DAo	3.68 (1.17; 6.18)	0.004	3.49 (1.01; 5.97)	0.006	3.42 (0.99; 5.85)	0.006
	LV Ejection fraction					
AAo	−0.44 (−1.37; 0.49)	0.349	−0.35 (−1.31; 0.61)	0.471	−0.49 (−1.45; 0.48)	0.322
DAo	−0.78 (−1.79; 0.23)	0.130	−0.75 (−1.79; 0.29)	0.156	−0.76 (−1.8; 0.27)	0.149
	LV Peak ejection rate					
AAo	18.3 (2.3; 34.2)	0.024	25.5 (9.7; 41.3)	0.002	23.6 (7.7; 39.4)	0.004
DAo	24.5 (7.2; 41.8)	0.006	30.7 (13.6; 47.8)	<0.001	30.6 (13.6; 47.6)	<0.001
	LV early diastolic rate					
AAo	2.2 (−11.2; 15.6)	0.747	9.9 (−3.3; 23.1)	0.141	8.46 (−4.81; 21.7)	0.211
DAo	11.3 (−3.26; 25.8)	0.128	18.06 (3.8; 32.3)	0.013	18.1 (3.92; 32.3)	0.013
	LV Late diastolic rate					
AAo	2.13 (−14.9; 19.2)	0.807	4.19 (−13.4; 21.8)	0.641	2.17 (−15.3; 19.6)	0.808
DAo	18.1 (−0.33; 36.7)	0.054	19.5 (0.49; 38.6)	0.044	20.3 (1.56; 39.1)	0.034
	LV Mass					
AAo	10.8 (7.84; 13.8)	<0.001	8.29 (5.49; 11.09)	<0.001	7.99 (5.17; 10.8)	<0.001
DAo	11.4 (8.15; 14.7)	<0.001	8.62 (5.57; 11.6)	<0.001	8.41 (5.36; 11.4)	<0.001
	RV End–diastolic Volume					
AAo	3.39 (−0.82; 7.6)	0.115	3.39 (−0.85; 7.63)	0.117	2.79 (−1.46; 7.04)	0.197
DAo	6.62 (2.07; 11.1)	0.004	6.56 (1.99; 11.1)	0.005	6.45 (1.9; 10.9)	0.006
	RV End–systolic Volume					
AAo	1.43 (−1.28; 4.14)	0.299	1.53 (−1.26; 4.31)	0.282	1.33 (−1.48; 4.14)	0.352
DAo	3.75 (0.83; 6.68)	0.012	3.99 (0.99; 6.99)	0.009	3.9 (0.89; 6.91)	0.011
	RV Stroke Volume					
AAo	1.89 (−0.36; 4.15)	0.099	1.8 (−0.45; 4.06)	0.116	1.41 (−0.83; 3.64)	0.218
DAo	2.80 (0.35; 5.25)	0.025	2.50 (0.06; 4.94)	0.044	2.49 (0.08; 4.89)	0.043
	RV Ejection fraction					
AAo	0.11 (−0.69; 0.92)	0.783	0.08 (−0.76; 0.91)	0.855	0.01 (−0.83; 0.85)	0.983
DAo	−0.25 (−1.13; 0.62)	0.568	−0.41 (−1.32; 0.49)	0.369	−0.4 (−1.3; 0.51)	0.391

ß-estimates given with 95% CI from linear regression analysis. The model 1 = adjusted for sex and age; model 2 = model 1 + BMI, smoking status, diabetes status, systolic and diastolic pressure; model 3 = model 2 + cholesterol, triglycerides, hypertension medication, and lipid lowering medication. CI = 95% confidence interval; SD = standard deviation. Abbreviation: AAo = ascending aorta; BMI = body mass index; DAo = descending aorta; HDL = high-density lipoprotein; LDL = low-density lipoprotein, LV = left ventricle; RV = right ventricle.

**Table 3:** Association between diameter of the pulmonary artery and ventricle parameters.

Diameter Per SD	Model 1	*p* Value	Model 2	*p* Value	Model 3	*p* Value
	LV End–diastolic Volume					
MPA	6.3 (3.16; 9.44)	<0.001	5.26 (2.09; 8.43)	0.001	4.81 (1.61; 8)	0.003
RPA	5.07 (1.79; 8.35)	0.003	5.06 (1.83; 8.3)	0.002	4.51 (1.23; 7.78)	0.007
LPA	10.5 (7.43; 13.6)	<0.001	9.74 (6.65; 12.8)	<0.001	9.09 (5.91; 12.2)	<0.001
	LV End–systolic Volume					
MPA	2.07 (0.25; 3.89)	0.026	1.73 (−0.14; 3.61)	0.069	1.79 (−0.11; 3.69)	0.065
RPA	1.36 (−0.53; 3.26)	0.159	1.19 (−0.72; 3.11)	0.220	1.20 (−0.76; 3.15)	0.229
LPA	3.4 (1.53; 5.27)	<0.001	3.24 (1.37; 5.12)	0.001	3.32 (1.38; 5.26)	0.001
	LV Stroke Volume					
MPA	4.27 (2.28; 6.26)	<0.001	3.56 (1.57; 5.56)	<0.001	3.06 (1.08; 5.04)	0.003
RPA	3.73 (1.65; 5.8)	<0.001	3.89 (1.86; 5.91)	<0.001	3.32 (1.30; 5.35)	0.001
LPA	7.18 (5.2; 9.15)	<0.001	6.52 (4.58; 8.45)	<0.001	5.79 (3.82; 7.76)	<0.001
	LV Ejection fraction					
MPA	−0.25 (−1.07; 0.56)	0.543	−0.29 (−1.13; 0.55)	0.501	−0.46 (−1.31; 0.39)	0.287
RPA	0.05 (−0.8; 0.89)	0.914	0.11 (−0.75; 0.96)	0.810	−0.04 (−0.91; 0.83)	0.932
LPA	−0.17 (−1.02; 0.68)	0.7	−0.23 (−1.09; 0.62)	0.592	−0.48 (−1.36; 0.39)	0.279
	LV Peak ejection rate					
MPA	23.5 (9.66; 37.4)	0.001	24.0 (10.1; 37.8)	0.001	21.6 (7.71; 35.6)	0.002
RPA	19.9 (5.51; 34.4)	0.007	23.8 (9.7; 37.9)	0.001	22.0 (7.74; 36.3)	0.003
LPA	42.1 (28.2; 56.06)	<0.001	40.0 (26.4; 53.7)	<0.001	37.7 (23.7; 51.7)	<0.001
	LV Early diastolic rate					
MPA	18.1 (6.48; 29.7)	0.002	19.6 (8.23; 31.1)	0.001	17.9 (6.37; 29.5)	0.002
RPA	13.4 (1.36; 25.6)	0.029	17.7 (6.03; 29.4)	0.003	16.4 (4.58; 28.3)	0.007
LPA	32.1 (20.4; 43.9)	<0.001	30.8 (19.5; 42.2)	<0.001	29.0 (17.3; 40.7)	<0.001
	LV Late diastolic rate					
MPA	17.8 (3.00; 32.7)	0.019	15.6 (0.25; 31.1)	0.046	11.9 (−3.45; 27.3)	0.128
RPA	17.5 (2.07; 32.9)	0.026	17.3 (1.59; 33.0)	0.031	14.03 (−1.73; 29.8)	0.081
LPA	32.1 (20.4; 43.9)	<0.001	30.8 (19.5; 42.2)	<0.001	29.0 (17.3; 40.7)	<0.001
	LV Mass					
MPA	5.41 (2.65; 8.17)	<0.001	3.96 (1.42; 6.51)	0.002	3.9 (1.33; 6.47)	0.003
RPA	5.14 (2.27; 8.01)	<0.001	3.9 (1.30; 6.49)	0.003	3.92 (1.29; 6.56)	0.004
LPA	5.09 (2.21; 7.97)	0.001	4.4 (1.82; 6.98)	0.001	4.15 (1.5; 6.8)	0.002
	RV End–diastolic Volume					
MPA	9.15 (5.57; 12.7)	<0.001	7.94 (4.3; 11.5)	<0.001	7.54 (3.87; 11.2)	<0.001
RPA	8.87 (5.14; 12.6)	<0.001	8.9 (5.3; 12.6)	<0.001	8.39 (4.65; 12.1)	<0.001
LPA	14.1 (10.5; 17.6)	<0.001	13.2 (9.78; 16.8)	<0.001	12.8 (9.21; 16.4)	<0.001
	RV End–systolic Volume					
MPA	5.0 (2.68; 7.32)	<0.001	4.59 (2.19; 6.98)	<0.001	4.62 (2.19; 7.05)	<0.001
RPA	4.72 (2.31; 7.14)	<0.001	4.82 (2.38; 7.26)	<0.001	4.71 (2.22; 7.2)	<0.001
LPA	6.99 (4.63; 9.34)	<0.001	6.74 (4.37; 9.12)	<0.001	6.84 (4.39; 9.28)	<0.001
	RV Stroke Volume					
MPA	4.09 (2.15; 6.03)	<0.001	3.29 (1.33; 5.24)	0.001	2.85 (0.9; 4.81)	0.004
RPA	4.05 (2.03; 6.06)	<0.001	4.08 (2.10; 6.05)	<0.001	3.58 (1.59; 5.57)	<0.001
LPA	7.1 (5.18; 9.02)	<0.001	6.48 (4.6; 8.37)	<0.001	5.92 (3.99; 7.86)	<0.001
	RV Ejection fraction					
MPA	−0.5 (−1.2; 0.2)	0.163	−0.63 (−1.36; 0.11)	0.093	−0.78 (−1.52; −0.04)	0.038
RPA	−0.39 (−1.12; 0.34)	0.296	−0.41 (−1.16; 0.33)	0.276	−0.53 (−1.29; 0.23)	0.170
LPA	−0.21 (−0.94; 0.53)	0.579	−0.34 (−1.09; 0.4)	0.365	−0.55 (−1.31; 0.21)	0.156

ß-estimates given with 95% CI from linear regression analysis. The model 1 = adjusted for sex and age; model 2 = model 1 + BMI, smoking status, diabetes status, systolic and diastolic pressure; model 3 = model 2 + cholesterol, triglycerides, hypertension medication, and lipid lowering medication. CI = 95% confidence interval; SD = standard deviation. Abbreviation: BMI = body mass index; HDL = high-density lipoprotein; LDL = low-density; lipoprotein, LV = left ventricle; RV = right ventricle; MPA = main pulmonary artery; RPA = right pulmonary artery; LPA = left pulmonary artery.

**Table 4:** Association between Ratio of MPA/Ao and ventricle parameters.

Ratio Per SD	Model 1	*p* Value	Model 2	*p* Value	Model 3	*p* Value
	LV End–diastolic Volume					
MPA/Ao	2.18 (−1.31; 5.68)	0.220	1.25 (−2.23; 4.73)	0.48	1.10 (−2.37; 4.57)	0.533
	LV End–systolic Volume					
MPA//Ao	0.01 (−1.99; 2.01)	0.994	−0.21 (−2.24; 1.83)	0.841	−0.18 (−2.23; 1.88)	0.866
	LV Stroke Volume					
MPA/Ao	2.22 (0.01; 4.44)	0.049	1.51 (−0.68; 3.7)	0.177	1.33 (−0.82; 3.48)	0.225
	LV Ejection fraction					
MPA/Ao	0.13 (−0.76; 1.02)	0.777	0.05 (−0.86; 0.96)	0.916	−0.02 (−0.93; 0.9)	0.971
	LV Peak ejection rate					
MPA/Ao	8.1 (−7.17; 23.5)	0.295	2.61 (−12.6; 17.8)	0.737	1.46 (−13.7; 16.6)	0.85
	LV Early diastolic rate					
MPA/Ao	16.3 (3.61; 29.0)	0.012	11.21 (−1.33; 23.7)	0.080	10.2 (−2.29; 22.8)	0.109
	LV Late diastolic rate					
MPA/Ao	14.5 (−1.79; 30.8)	0.081	10.5 (−6.27; 27.2)	0.219	8.47 (−8.1; 25.0)	0.315
	LV Mass					
MPA/Ao	−3.46 (−6.51;−0.41)	0.026	−2.59 (−5.37; 0.19)	0.068	−2.51 (−5.3; 0.27)	0.076
	RV End–diastolic Volume					
MPA/Ao	5.82 (1.82; 9.83)	0.004	4.57 (0.56; 8.59)	0.026	4.48 (0.47; 8.49)	0.029
	RV End–systolic Volume					
MPA/Ao	3.30 (0.73; 5.87)	0.012	2.73 (0.09; 5.36)	0.042	2.82 (0.17; 5.46)	0.037
	RV Stroke Volume					
MPA/Ao	2.52 (0.36; 4.67)	0.022	1.84 (−0.30; 3.98)	0.092	1.65 (−0.47; 3.77)	0.126
	RV Ejection fraction					
MPA/Ao	−0.45 (−1.21; 0.32)	0.255	−0.50 (−1.29; 0.29)	0.216	−0.59 (−1.38; 0.21)	0.148

ß-estimates given with 95% CI from linear regression analysis. The model 1 = adjusted for sex and age; model 2 = model 1 + BMI, smoking status, diabetes status, systolic and diastolic pressure; model 3 = model 2 + cholesterol, triglycerides, hypertension medication, and lipid lowering medication. CI = 95% confidence interval; SD = standard deviation. Abbreviation: Ao = aorta; BMI = body mass index; LV = left ventricle; MPA = main pulmonary artery; RV = right ventricle.

## Data Availability

The informed consent given by KORA study participants does not cover data posting in public databases. However, data are available upon request by means of a project agreement. Requests should be sent to kora.passt@helmholtz-muenchen.de and are subject to approval by the KORA Board.

## Supplementary Materials

The following supporting information can be downloaded at: https://www.mdpi.com/article/10.3390/jpm12060889/s1, Tables S1–S3: Association between diameters of the aorta/pulmonary artery system/ratio of MPA/Ao and ventricle function parameters according to gender (females). Tables S4–S6: Association between diameters of the aorta/the pulmonary artery system/ratio of MPA/Ao and ventricle function parameters according to gender (males).
