# Supplementary material for: Association between Large Arteries Diameter and Heart Function in Subjects Free of Cardiovascular Diseases
Source: J Pers Med. 2022 May 28;12(6):889. doi: 10.3390/jpm12060889 (PMC9225228; doi:10.3390/jpm12060889)
Supplement: Supplementary file 1 [file jpm-12-00889-s001.zip › jpm-1681037-supplementary.pdf]

Table S1. Association between diameters of the aorta and ventricle function parameters according to gender.

Diameter per SD FEMALE	Model 1	p-value	Model 2	p-value	Model 3	p-value
**LV End–diastolic Volume**						
AAo	1.28 (-3.42; 5.98)	0.592	-0.19 (-5.14; 4.75)	0.938	0.08 (-4.86; 5.03)	0.973
ADo	5.03 (-0.21; 10.28)	0.06	3.06 (-2.32; 8.44)	0.263	2.57 (-2.78; 7.91)	0.344
**LV End–systolic Volume**						
AAo	0.63 (-2.08; 3.34)	0.648	-0.25 (-3.1; 2.61)	0.865	0.06 (-2.82; 2.95)	0.965
ADo	0.44 (-2.62; 3.49)	0.779	-0.12 (-3.23; 3)	0.94	-0.06 (-3.18; 3.07)	0.972
**LV Stroke Volume**						
AAo	0.65 (-2.41; 3.72)	0.675	0.06 (-3.12; 3.24)	0.972	0.02 (-3.06; 3.11)	0.988
ADo	4.55 (1.17; 7.93)	0.009	3.12 (-0.31; 6.55)	0.074	2.57 (-0.74; 5.88)	0.128
**LV Ejection fraction**						
AAo	-0.09 (-1.33; 1.16)	0.889	0 (-1.32; 1.32)	0.999	-0.19 (-1.49; 1.12)	0.779
ADo	0.66 (-0.74; 2.06)	0.351	0.65 (-0.79; 2.09)	0.375	0.47 (-0.94; 1.88)	0.512
**LV Peak ejection rate**						
AAo	13.16 (-6.52; 32.83)	0.188	19.59 (-1.15; 40.32)	0.064	18.34 (-2.52; 39.2)	0.084
ADo	23.58 (1.6; 45.56)	0.036	21.27 (-1.37; 43.92)	0.065	19 (-3.63; 41.63)	0.099
**LV early diastolic rate**						
AAo	-2.82 (-21.18; 15.55)	0.762	7.94 (-11.08; 26.96)	0.411	6.73 (-12.11; 25.57)	0.481
ADo	10.02 (-10.62; 30.66)	0.339	11.76 (-8.97; 32.49)	0.264	8.96 (-11.44; 29.36)	0.386
**LV Late diastolic rate**						
AAo	-14.21 (-38.8; 10.38)	0.255	-16.92 (-43.69; 9.84)	0.213	-22.9 (-49.46; 3.66)	0.09
ADo	27.81 (0.34; 55.28)	0.047	28.87 (-0.12; 57.85)	0.051	27.35 (-1.37; 56.07)	0.062
**LV Mass**						
AAo	8.63 (4.33; 12.93)	<0.001	4.24 (0.37; 8.11)	0.032	4.3 (0.44; 8.17)	0.029
ADo	8.42 (3.51; 13.34)	0.001	3.97 (-0.28; 8.21)	0.067	3.68 (-0.53; 7.9)	0.086
**RV End–diastolic Volume**						
AAo	1.36 (-4.32; 7.04)	0.637	0.7 (-5.19; 6.6)	0.814	1.03 (-4.93; 6.98)	0.733
ADo	5.43 (-0.91; 11.78)	0.093	2.9 (-3.52; 9.32)	0.373	2.67 (-3.77; 9.11)	0.413
**RV End–systolic Volume**						
AAo	0.48 (-3.05; 4)	0.79	0.28 (-3.56; 4.11)	0.887	0.61 (-3.24; 4.47)	0.753
ADo	1.35 (-2.61; 5.32)	0.501	0.54 (-3.64; 4.73)	0.797	0.7 (-3.48; 4.87)	0.741
**RV Stroke Volume**						
AAo	0.76 (-2.4; 3.92)	0.636	0.28 (-2.9; 3.47)	0.86	0.28 (-2.9; 3.45)	0.864
ADo	4.01 (0.5; 7.51)	0.025	2.28 (-1.17; 5.73)	0.194	1.9 (-1.53; 5.33)	0.275
**RV Ejection fraction**						
AAo	0.19 (-1; 1.38)	0.757	0.12 (-1.19; 1.42)	0.861	-0.03 (-1.32; 1.25)	0.962
ADo	0.72 (-0.62; 2.06)	0.289	0.48 (-0.95; 1.9)	0.51	0.31 (-1.08; 1.71)	0.657

ß-estimates given with 95% CI from linear regression analysis. The model 1 = adjusted for sex and age; model 2 = model 1 + BMI, smoking status, diabetes status, systolic and diastolic pressure; model 3 = model 2 + cholesterol, triglycerides, hypertension medication, and lipid lowering medication. CI = 95% confidence interval; SD = standard deviation. Abbreviation: AAo= Ascending aorta; ADo= Descending aorta; BMI = body mass index; HDL = high-density lipoprotein; LDL = low-density lipoprotein, LV = left ventricle; RV = right ventricle

Table S2. Association between diameters of the pulmonary artery system and ventricle function parameters according to gender.

Diameter per SD FEMALE	Model 1	p-value	Model 2	p-value	Model 3	p-value
**LV End–diastolic Volume**						
MPA	7.62 (3.82; 11.42)	<0.001	6.82 (2.57; 11.07)	0.002	6.65 (2.37; 10.93)	0.003
RPA	5.26 (1.08; 9.43)	0.014	3.79 (-0.45; 8.03)	0.08	3.63 (-0.62; 7.87)	0.093
LPA	9.85 (5.91; 13.79)	<0.001	9.2 (5.02; 13.37)	<0.001	8.27 (3.94; 12.6)	<0.001
**LV End–systolic Volume**						
MPA	2.28 (0.01; 4.56)	0.049	2.53 (0.03; 5.03)	0.047	2.86 (0.33; 5.4)	0.027
RPA	1.08 (-1.37; 3.53)	0.386	0.37 (-2.1; 2.85)	0.766	0.4 (-2.1; 2.9)	0.752
LPA	3.15 (0.75; 5.55)	0.011	3.13 (0.62; 5.64)	0.015	3.14 (0.54; 5.74)	0.018
**LV Stroke Volume**						
MPA	5.33 (2.88; 7.79)	<0.001	4.29 (1.55; 7.02)	0.002	3.79 (1.1; 6.47)	0.006
RPA	4.19 (1.5; 6.89)	0.003	3.44 (0.74; 6.14)	0.013	3.23 (0.62; 5.85)	0.016
LPA	6.7 (4.15; 9.25)	<0.001	6.06 (3.39; 8.74)	<0.001	5.14 (2.44; 7.84)	<0.001
**LV Ejection fraction**						
MPA	-0.23 (-1.28; 0.83)	0.674	-0.52 (-1.69; 0.66)	0.386	-0.86 (-2.02; 0.3)	0.146
RPA	0.41 (-0.71; 1.54)	0.469	0.6 (-0.55; 1.74)	0.303	0.58 (-0.55; 1.71)	0.31
LPA	-0.14 (-1.26; 0.99)	0.813	-0.24 (-1.43; 0.95)	0.69	-0.5 (-1.7; 0.7)	0.411
**LV Peak ejection rate**						
MPA	35.23 (19.44; 51.01)	<0.001	35.36 (17.65; 53.08)	<0.001	32.72 (14.67; 50.77)	<0.001
RPA	24.73 (7.25; 42.21)	0.006	24.62 (6.89; 42.35)	0.007	25.29 (7.5; 43.07)	0.006
LPA	29.73 (12.5; 46.96)	0.001	30.72 (12.54; 48.89)	0.001	28.68 (9.89; 47.47)	0.003
**LV early diastolic rate**						
MPA	26.98 (12.02; 41.94)	<0.001	30.95 (14.8; 47.11)	<0.001	28.11 (11.9; 44.32)	0.001
RPA	15.29 (-1.18; 31.75)	0.069	17.9 (1.64; 34.16)	0.031	17.19 (1.08; 33.3)	0.037
LPA	26.39 (10.35; 42.44)	0.001	30.24 (13.86; 46.63)	<0.001	26.99 (10.24; 43.75)	0.002
**LV Late diastolic rate**						
MPA	3.34 (-17.63; 24.32)	0.753	1.85 (-22.1; 25.81)	0.879	-3.12 (-27.16; 20.92)	0.798
RPA	8.16 (-14.19; 30.52)	0.472	5.43 (-17.89; 28.75)	0.646	5.97 (-17.3; 29.25)	0.613
LPA	18.33 (-3.81; 40.48)	0.104	19.12 (-4.89; 43.12)	0.118	22.54 (-1.87; 46.95)	0.07
**LV Mass**						
MPA	8.06 (4.45; 11.67)	<0.001	5.48 (2.09; 8.86)	0.002	5.22 (1.81; 8.63)	0.003
RPA	5.45 (1.44; 9.45)	0.008	2.5 (-0.89; 5.89)	0.147	2.74 (-0.64; 6.13)	0.111
LPA	8.2 (4.33; 12.07)	<0.001	4.68 (1.23; 8.13)	0.008	4.22 (0.67; 7.77)	0.02
**RV End–diastolic Volume**						
MPA	11.83 (7.41; 16.25)	<0.001	10.13 (5.17; 15.1)	<0.001	10.6 (5.58; 15.62)	<0.001
RPA	9.41 (4.49; 14.32)	<0.001	7.98 (3.05; 12.92)	0.002	7.56 (2.55; 12.56)	0.003
LPA	12.75 (8.06; 17.45)	<0.001	11.51 (6.57; 16.45)	<0.001	11.5 (6.38; 16.62)	<0.001
**RV End–systolic Volume**						
MPA	5.82 (2.98; 8.66)	<0.001	5.83 (2.57; 9.1)	0.001	6.58 (3.32; 9.85)	<0.001
RPA	4.63 (1.52; 7.73)	0.004	4.26 (1.02; 7.51)	0.01	3.97 (0.69; 7.24)	0.018
LPA	5.59 (2.53; 8.65)	<0.001	5.43 (2.1; 8.76)	0.002	5.93 (2.53; 9.33)	0.001
**RV Stroke Volume**						
MPA	5.92 (3.41; 8.42)	<0.001	4.19 (1.45; 6.93)	0.003	3.91 (1.14; 6.67)	0.006
RPA	4.61 (1.85; 7.37)	0.001	3.54 (0.85; 6.24)	0.01	3.42 (0.72; 6.11)	0.013
LPA	7.07 (4.45; 9.68)	<0.001	5.99 (3.31; 8.67)	<0.001	5.47 (2.7; 8.24)	<0.001
**RV Ejection fraction**						
MPA	-0.4 (-1.41; 0.61)	0.432	-1.01 (-2.15; 0.14)	0.086	-1.37 (-2.5; -0.25)	0.017
RPA	-0.39 (-1.47; 0.68)	0.47	-0.61 (-1.74; 0.52)	0.286	-0.53 (-1.64; 0.59)	0.351
LPA	0 (-1.08; 1.07)	0.993	-0.35 (-1.53; 0.82)	0.55	-0.66 (-1.84; 0.52)	0.272

ß-estimates given with 95% CI from linear regression analysis. The model 1 = adjusted for sex and age; model 2 = model 1 + BMI, smoking status, diabetes status, systolic and diastolic pressure; model 3 = model 2 + cholesterol, triglycerides, hypertension medication, and lipid lowering medication. CI = 95% confidence interval; SD = standard deviation. Abbreviation: BMI = body mass index; HDL = high-density lipoprotein; LDL = low-density lipoprotein, LV = left ventricle; MPA = main pulmonary artery; RV = right ventricle; Diam.= diameter; RPA= right pulmonary artery; LPA= left pulmonary artery

Table S3. Association between ratio of MPA/Ao and ventricle function parameters according to gender.

Diameter per SD FEMALE	Model 1	p-value	Model 2	p-value	Model 3	p-value
			**LV End–diastolic Volume**			
MPA/Ao	5.83 (1.84; 9.82)	0.004	5.91 (1.68; 10.15)	0.007	5.42 (1.2; 9.65)	0.012
			**LV End–systolic Volume**			
MPA/Ao	1.45 (-0.9; 3.8)	0.226	2.13 (-0.35; 4.61)	0.091	2.14 (-0.35; 4.64)	0.091
			**LV Stroke Volume**			
MPA/Ao	4.38 (1.8; 6.96)	0.001	3.77 (1.05; 6.49)	0.007	3.28 (0.64; 5.92)	0.015
			**LV Ejection fraction**			
MPA/Ao	-0.06 (-1.15; 1.03)	0.916	-0.31 (-1.47; 0.85)	0.598	-0.46 (-1.6; 0.67)	0.421
			**LV Peak ejection rate**			
MPA/Ao	20.88 (3.96; 37.79)	0.016	14.19 (-4.12; 32.5)	0.128	12.02 (-6.32; 30.36)	0.197
			**LV early diastolic rate**			
MPA/Ao	26.46 (11.04; 41.88)	0.001	20.34 (3.92; 36.75)	0.016	18.09 (1.86; 34.31)	0.029
			**LV Late diastolic rate**			
MPA/Ao	14.63 (-6.78; 36.03)	0.179	15.52 (-8; 39.04)	0.194	15.01 (-8.34; 38.36)	0.206
			**LV Mass**			
MPA/Ao	0.46 (-3.49; 4.41)	0.818	1.27 (-2.19; 4.72)	0.469	0.88 (-2.56; 4.32)	0.615
			**RV End–diastolic Volume**			
MPA/Ao	9.6 (4.9; 14.3)	<0.001	8.19 (3.19; 13.19)	0.002	8.14 (3.11; 13.16)	0.002
			**RV End–systolic Volume**			
MPA/Ao	4.79 (1.81; 7.76)	0.002	4.65 (1.37; 7.92)	0.006	4.92 (1.65; 8.19)	0.003
			**RV Stroke Volume**			
MPA/Ao	4.83 (2.19; 7.47)	<0.001	3.57 (0.83; 6.3)	0.011	3.24 (0.51; 5.96)	0.02
			**RV Ejection fraction**			
MPA/Ao	-0.44 (-1.48; 0.59)	0.401	-0.82 (-1.96; 0.32)	0.156	-1.01 (-2.12; 0.1)	0.074

ß-estimates given with 95% CI from linear regression analysis. The model 1 = adjusted for sex and age; model 2 = model 1 + BMI, smoking status, diabetes status, systolic and diastolic pressure; model 3 = model 2 + cholesterol, triglycerides, hypertension medication, and lipid lowering medication. CI = 95% confidence interval; SD = standard deviation. Ao = aorta; BMI = body mass index; HDL = high-density lipoprotein; LDL = low-density lipoprotein, LV = left ventricle; MPA = main pulmonary artery; RV = right ventricle

Table S4. Association between diameters of the aorta and ventricle function parameters according to gender.

Diameter per SD MALE	Model 1	p-value	Model 2	p-value	Model 3	p-value
			**LV End–diastolic Volume**			
AAo	8.07 (2.81; 13.34)	0.003	7.75 (2.52; 12.98)	0.004	7.09 (1.73; 12.44)	0.01
ADo	9.13 (3.54; 14.71)	0.001	10.12 (4.62; 15.62)	<0.001	10.14 (4.61; 15.67)	<0.001
			**LV End–systolic Volume**			
AAo	4 (0.98; 7.02)	0.01	3.76 (0.67; 6.85)	0.017	3.78 (0.61; 6.96)	0.02
ADo	6.11 (2.95; 9.26)	<0.001	6.56 (3.36; 9.75)	<0.001	6.57 (3.33; 9.82)	<0.001
			**LV Stroke Volume**			
AAo	4.04 (0.7; 7.38)	0.018	3.95 (0.62; 7.29)	0.021	3.25 (-0.12; 6.62)	0.058
ADo	3.01 (-0.57; 6.59)	0.098	3.55 (-0.02; 7.11)	0.051	3.55 (0.02; 7.09)	0.049
			**LV Ejection fraction**			
AAo	-0.7 (-2.05; 0.64)	0.305	-0.58 (-1.98; 0.82)	0.417	-0.75 (-2.18; 0.67)	0.3
ADo	-1.79 (-3.2; -0.38)	0.013	-1.79 (-3.26; -0.32)	0.017	-1.79 (-3.27; -0.31)	0.018
			**LV Peak ejection rate**			
AAo	22.22 (-1.72; 46.17)	0.069	27.07 (3.65; 50.49)	0.024	25.42 (1.46; 49.39)	0.038
ADo	25.19 (-0.26; 50.64)	0.052	33.44 (8.65; 58.23)	0.008	33.64 (8.65; 58.63)	0.009
			**LV early diastolic rate**			
AAo	5.98 (-13.27; 25.23)	0.541	10.3 (-8.26; 28.85)	0.275	9.1 (-9.84; 28.04)	0.345
ADo	12.28 (-8.15; 32.7)	0.237	17.7 (-1.94; 37.34)	0.077	17.71 (-2.07; 37.48)	0.079
			**LV Late diastolic rate**			
AAo	14.52 (-9.16; 38.2)	0.228	18.45 (-5.95; 42.86)	0.137	19 (-5.63; 43.64)	0.13
ADo	11.65 (-13.6; 36.89)	0.364	13.24 (-12.81; 39.29)	0.317	15.09 (-10.87; 41.04)	0.253
			**LV Mass**			
AAo	12.54 (8.41; 16.68)	<0.001	11.79 (7.81; 15.77)	<0.001	11.54 (7.45; 15.64)	<0.001
ADo	13.32 (8.92; 17.72)	<0.001	12.44 (8.19; 16.68)	<0.001	12.25 (7.96; 16.54)	<0.001
			**RV End–diastolic Volume**			
AAo	4.94 (-1.14; 11.03)	0.11	4.03 (-2.13; 10.18)	0.198	3.03 (-3.25; 9.3)	0.342
ADo	7.37 (0.94; 13.8)	0.025	8.34 (1.87; 14.8)	0.012	8.34 (1.84; 14.83)	0.012
			**RV End–systolic Volume**			
AAo	2.15 (-1.82; 6.12)	0.287	1.5 (-2.58; 5.58)	0.469	1.07 (-3.13; 5.26)	0.616
ADo	5.38 (1.21; 9.55)	0.012	6.1 (1.84; 10.36)	0.005	6.01 (1.69; 10.32)	0.007
			**RV Stroke Volume**			
AAo	2.78 (-0.41; 5.96)	0.087	2.52 (-0.72; 5.77)	0.126	1.97 (-1.31; 5.25)	0.237
ADo	1.92 (-1.48; 5.32)	0.267	2.18 (-1.28; 5.64)	0.215	2.28 (-1.16; 5.72)	0.192
			**RV Ejection fraction**			
AAo	0.07 (-1.02; 1.16)	0.902	0.18 (-0.97; 1.34)	0.754	0.13 (-1.05; 1.3)	0.83
ADo	-0.95 (-2.1; 0.21)	0.108	-1.05 (-2.27; 0.17)	0.091	-1 (-2.22; 0.23)	0.111

ß-estimates given with 95% CI from linear regression analysis. The model 1 = adjusted for sex and age; model 2 = model 1 + BMI, smoking status, diabetes status, systolic and diastolic pressure; model 3 = model 2 + cholesterol, triglycerides, hypertension medication, and lipid lowering medication. CI = 95% confidence interval; SD = standard deviation. Abbreviation: AAo= Ascending aorta; ADo= Descending aorta; BMI = body mass index; HDL = high-density lipoprotein; LDL = low-density lipoprotein, LV = left ventricle; RV = right ventricle

Table S5. Association between diameter of the pulmonary artery system and ventricle function parameters according to gender.

Diameter per SD MALE	Model 1	p-value	Model 2	p-value	Model 3	p-value
**LV End–diastolic Volume**						
MPA	5.1 (0.27; 9.92)	0.038	4.48 (-0.3; 9.27)	0.066	3.96 (-0.94; 8.86)	0.112
RPA	5.22 (0.32; 10.12)	0.037	6.51 (1.76; 11.25)	0.007	6.1 (1.19; 11.02)	0.015
LPA	11.14 (6.46; 15.82)	<0.001	9.57 (5.01; 14.13)	<0.001	9.22 (4.42; 14.01)	<0.001
**LV End–systolic Volume**						
MPA	1.89 (-0.88; 4.66)	0.181	1.37 (-1.45; 4.19)	0.34	1.48 (-1.43; 4.39)	0.316
RPA	1.62 (-1.2; 4.44)	0.259	2.02 (-0.8; 4.85)	0.158	2.27 (-0.67; 5.21)	0.129
LPA	3.6 (0.83; 6.38)	0.011	2.75 (-0.02; 5.51)	0.051	3.07 (0.16; 5.98)	0.039
**LV Stroke Volume**						
MPA	3.28 (0.25; 6.31)	0.034	3.18 (0.17; 6.2)	0.039	2.55 (-0.51; 5.6)	0.101
RPA	3.62 (0.54; 6.69)	0.021	4.5 (1.51; 7.49)	0.003	3.85 (0.78; 6.91)	0.014
LPA	7.57 (4.66; 10.48)	<0.001	6.85 (4.01; 9.7)	<0.001	6.17 (3.2; 9.14)	<0.001
**LV Ejection fraction**						
MPA	-0.32 (-1.53; 0.9)	0.61	-0.16 (-1.42; 1.11)	0.804	-0.38 (-1.67; 0.91)	0.562
RPA	-0.16 (-1.4; 1.08)	0.805	-0.19 (-1.46; 1.08)	0.766	-0.49 (-1.79; 0.82)	0.465
LPA	-0.18 (-1.42; 1.06)	0.774	0.05 (-1.2; 1.3)	0.939	-0.3 (-1.6; 1.01)	0.655
**LV Peak ejection rate**						
MPA	13.58 (-8.19; 35.34)	0.22	11.74 (-9.62; 33.1)	0.28	9.7 (-12.2; 31.6)	0.383
RPA	16.56 (-5.53; 38.66)	0.141	21.15 (-0.11; 42.41)	0.051	18.96 (-3.1; 41.01)	0.092
LPA	51.81 (30.94; 72.69)	<0.001	42.76 (22.54; 62.97)	<0.001	41.79 (20.51; 63.07)	<0.001
**LV early diastolic rate**						
MPA	10.64 (-6.72; 28)	0.228	8.18 (-8.57; 24.94)	0.337	7.61 (-9.53; 24.75)	0.382
RPA	12.14 (-5.5; 29.78)	0.176	15.12 (-1.58; 31.82)	0.076	14.48 (-2.79; 31.75)	0.1
LPA	36.62 (19.75; 53.5)	<0.001	28.21 (12.14; 44.28)	0.001	28.34 (11.51; 45.17)	0.001
**LV Late diastolic rate**						
MPA	30.34 (9.28; 51.4)	0.005	30.82 (9.13; 52.5)	0.006	26.71 (4.62; 48.79)	0.018
RPA	25.3 (3.73; 46.86)	0.022	27.62 (5.78; 49.46)	0.013	22.12 (-0.36; 44.61)	0.054
LPA	44.38 (23.52; 65.24)	<0.001	39.64 (18.55; 60.74)	<0.001	35.8 (13.77; 57.83)	0.002
**LV Mass**						
MPA	2.99 (-1.06; 7.05)	0.147	3.92 (0.05; 7.8)	0.047	4.01 (0.04; 7.98)	0.048
RPA	5.34 (1.26; 9.41)	0.011	5.48 (1.64; 9.33)	0.005	5.73 (1.76; 9.71)	0.005
LPA	2.71 (-1.41; 6.83)	0.195	4.28 (0.46; 8.1)	0.028	4.23 (0.22; 8.23)	0.039
**RV End–diastolic Volume**						
MPA	6.82 (1.37; 12.27)	0.014	5.64 (0.12; 11.16)	0.045	4.9 (-0.74; 10.55)	0.088
RPA	8.74 (3.25; 14.23)	0.002	9.76 (4.35; 15.17)	<0.001	9.23 (3.62; 14.83)	0.001
LPA	15.23 (10.06; 20.41)	<0.001	13.86 (8.74; 18.99)	<0.001	13.47 (8.07; 18.86)	<0.001
**RV End–systolic Volume**						
MPA	4.32 (0.76; 7.87)	0.018	3.37 (-0.28; 7.03)	0.07	3.31 (-0.45; 7.07)	0.084
RPA	4.86 (1.26; 8.46)	0.008	5.2 (1.58; 8.82)	0.005	5.29 (1.53; 9.05)	0.006
LPA	8.07 (4.6; 11.54)	<0.001	7.31 (3.83; 10.79)	<0.001	7.5 (3.83; 11.17)	<0.001
**RV Stroke Volume**						
MPA	2.46 (-0.42; 5.34)	0.094	2.24 (-0.69; 5.16)	0.133	1.55 (-1.41; 4.52)	0.303
RPA	3.84 (0.94; 6.74)	0.01	4.53 (1.66; 7.41)	0.002	3.89 (0.93; 6.85)	0.01
LPA	7.14 (4.38; 9.9)	<0.001	6.52 (3.77; 9.27)	<0.001	5.92 (3.05; 8.8)	<0.001
**RV Ejection fraction**						
MPA	-0.62 (-1.61; 0.36)	0.212	-0.44 (-1.48; 0.6)	0.407	-0.65 (-1.71; 0.41)	0.228
RPA	-0.3 (-1.31; 0.7)	0.552	-0.19 (-1.23; 0.86)	0.725	-0.44 (-1.51; 0.64)	0.424
LPA	-0.35 (-1.36; 0.65)	0.487	-0.34 (-1.36; 0.69)	0.516	-0.63 (-1.7; 0.44)	0.249

ß-estimates given with 95% CI from linear regression analysis. The model 1 = adjusted for sex and age; model 2 = model 1 + BMI, smoking status, diabetes status, systolic and diastolic pressure; model 3 = model 2 + cholesterol, triglycerides, hypertension medication, and lipid lowering medication. CI = 95% confidence interval; SD = standard deviation. Abbreviation: BMI = body mass index; HDL = high-density lipoprotein; LDL = low-density lipoprotein, LV = left ventricle; MPA = main pulmonary artery; RV = right ventricle; Diam.= diameter; RPA= right pulmonary artery; LPA= left pulmonary artery

Table S6. Association between ratio of MPA/Ao and ventricle function parameters according to gender.

Diameter per SD MALE	Model 1	p-value	Model 2	p-value	Model 3	p-value
			**LV End–diastolic Volume**			
MPA/Ao	-1.86 (-7.49; 3.77)	0.515	-2.15 (-7.63; 3.33)	0.44	-2.19 (-7.76; 3.37)	0.438
			**LV End–systolic Volume**			
MPA/Ao	-1.56 (-4.77; 1.64)	0.337	-1.85 (-5.06; 1.35)	0.255	-1.76 (-5.05; 1.53)	0.292
			**LV Stroke Volume**			
MPA/Ao	-0.2 (-3.74; 3.34)	0.913	-0.19 (-3.66; 3.28)	0.914	-0.32 (-3.8; 3.16)	0.856
			**LV Ejection fraction**			
MPA/Ao	0.3 (-1.11; 1.71)	0.677	0.36 (-1.07; 1.8)	0.619	0.27 (-1.19; 1.74)	0.711
			**LV Peak ejection rate**			
MPA/Ao	-5.58 (-30.8; 19.65)	0.663	-11.35 (-35.64; 12.94)	0.358	-12.35 (-37.08; 12.38)	0.326
			**LV early diastolic rate**			
MPA/Ao	5.47 (-14.64; 25.59)	0.592	-0.97 (-20.06; 18.12)	0.92	-0.96 (-20.37; 18.45)	0.922
			**LV Late diastolic rate**			
MPA/Ao	14.33 (-10.42; 39.09)	0.255	10.68 (-14.45; 35.8)	0.403	5.88 (-19.46; 31.21)	0.648
			**LV Mass**			
MPA/Ao	-7.87 (-12.45; -3.3)	0.001	-6.01 (-10.38; -1.65)	0.007	-5.77 (-10.22; -1.32)	0.011
			**RV End–diastolic Volume**			
MPA/Ao	1.67 (-4.72; 8.07)	0.606	1.11 (-5.23; 7.45)	0.73	1.06 (-5.37; 7.49)	0.745
			**RV End–systolic Volume**			
MPA/Ao	1.7 (-2.46; 5.86)	0.421	1.15 (-3.04; 5.34)	0.589	1.35 (-2.93; 5.64)	0.534
			**RV Stroke Volume**			
MPA/Ao	-0.06 (-3.41; 3.3)	0.973	-0.07 (-3.41; 3.28)	0.968	-0.34 (-3.7; 3.02)	0.842
			**RV Ejection fraction**			
MPA/Ao	-0.49 (-1.63; 0.65)	0.401	-0.35 (-1.53; 0.83)	0.555	-0.51 (-1.71; 0.69)	0.407

ß-estimates given with 95% CI from linear regression analysis. The model 1 = adjusted for sex and age; model 2 = model 1 + BMI, smoking status, diabetes status, systolic and diastolic pressure; model 3 = model 2 + cholesterol, triglycerides, hypertension medication, and lipid lowering medication. CI = 95% confidence interval; SD = standard deviation. Ao = aorta; BMI = body mass index; HDL = high-density lipoprotein; LDL = low-density lipoprotein, LV = left ventricle; MPA = main pulmonary artery; RV = right ventricle
